# Personal

**Published:** 1895-05

**Authors:** 


### Personal.

Dr. S. W. Wetmore and his wife, Mary B. Wetmore, M. D., formerly at 149 Franklin street, Buffalo, having spent the most of three winters in San Diego, Cal., have returned and built a new house, which they are now occupying, at 30 Woodlawn avenue, corner Otis place. Hours, 8 to 10 A. M.; 1 to 3 and 7 P. M. Telephone, Bryant 327.

---

Dr. H. G. Hopkins, formerly assistant physician at Willard State Hospital, announces his location at 220 Jersey street, Buffalo, where he will give special attention to nervous and mental diseases. Hours, 8 to 9 A. M.; 2 to 3 and 6.30 to 7.30 P. M.

---

Dr. John Parmenter, professor of anatomy and adjunct professor of clinical surgery in Buffalo University Medical College, has removed from 116 North Pearl street to 519 Franklin street, Buffalo.

---

Dr. F. W. Hinkel, clinical professor of laryngology in Buffalo University Medical College, has removed his office from 274 Delaware avenue to its former location, 305 Delaware avenue, Buffalo.

---

Dr. Charles E. Congdon, of 1034 Jefferson street, Buffalo, has returned from a six months' absence in Europe, and has again taken up his professional labors.

---

Dr. William G. Ring, of 364 Niagara street, Buffalo, who has been spending some time in Europe, has recently returned and resumed his professional work.

